# The responses of an anaerobic microorganism, *Yersinia intermedia* MASE-LG-1 to individual and combined simulated Martian stresses

**DOI:** 10.1371/journal.pone.0185178

**Published:** 2017-10-25

**Authors:** Kristina Beblo-Vranesevic, Maria Bohmeier, Alexandra K. Perras, Petra Schwendner, Elke Rabbow, Christine Moissl-Eichinger, Charles S. Cockell, Rüdiger Pukall, Pauline Vannier, Viggo T. Marteinsson, Euan P. Monaghan, Pascale Ehrenfreund, Laura Garcia-Descalzo, Felipe Gómez, Moustafa Malki, Ricardo Amils, Frédéric Gaboyer, Frances Westall, Patricia Cabezas, Nicolas Walter, Petra Rettberg

**Affiliations:** 1 Radiation Biology Department, Institute of Aerospace Medicine, German Aerospace Center (DLR), Cologne, Germany; 2 Department of Internal Medicine, Medical University of Graz, Graz, Austria; 3 Department of Microbiology and Archaea, University of Regensburg, Regensburg, Germany; 4 School of Physics and Astronomy, UK Center for Astrobiology, University of Edinburgh, Edinburgh, United Kingdom; 5 BioTechMed Graz, Graz, Austria; 6 German Collection of Microorganisms and Cell Cultures, Leibniz Institute DSMZ, Braunschweig, Germany; 7 MATIS—Prokaria, Reykjavík, Iceland; 8 Faculty of Food Science and Nutrition, University of Iceland, Reykjavík, Iceland; 9 Leiden Observatory, Universiteit Leiden, Leiden, Netherland; 10 Space Policy Institute, George Washington University, Washington DC, United States of America; 11 Instituto Nacional de Técnica Aeroespacial—Centro de Astrobiología (INTA-CAB), Madrid, Spain; 12 Universidad Autónoma de Madrid (UAM), Madrid, Spain; 13 Centre de Biophysique Moléculaire, Centre National de la Recherche Scientifique (CNRS), Orléans, France; 14 European Science Foundation (ESF), Strasbourg, France; ENEA Centro Ricerche Casaccia, ITALY

## Abstract

The limits of life of aerobic microorganisms are well understood, but the responses of anaerobic microorganisms to individual and combined extreme stressors are less well known. Motivated by an interest in understanding the survivability of anaerobic microorganisms under Martian conditions, we investigated the responses of a new isolate, *Yersinia intermedia* MASE-LG-1 to individual and combined stresses associated with the Martian surface. This organism belongs to an adaptable and persistent genus of anaerobic microorganisms found in many environments worldwide. The effects of desiccation, low pressure, ionizing radiation, varying temperature, osmotic pressure, and oxidizing chemical compounds were investigated. The strain showed a high tolerance to desiccation, with a decline of survivability by four orders of magnitude during a storage time of 85 days. Exposure to X-rays resulted in dose-dependent inactivation for exposure up to 600 Gy while applied doses above 750 Gy led to complete inactivation. The effects of the combination of desiccation and irradiation were additive and the survivability was influenced by the order in which they were imposed. Ionizing irradiation and subsequent desiccation was more deleterious than vice versa. By contrast, the presence of perchlorates was not found to significantly affect the survival of the *Yersinia* strain after ionizing radiation. These data show that the organism has the capacity to survive and grow in physical and chemical stresses, imposed individually or in combination that are associated with Martian environment. Eventually it lost its viability showing that many of the most adaptable anaerobic organisms on Earth would be killed on Mars today.

## Introduction

Considering the planetary bodies in our solar system in terms of their environmental conditions, Mars is the most Earth-like planet, at least in its early history [1–3]. A crucial question remains as to whether habitable, potentially inhabited, environments exist or have existed on Mars. Assessing the potential habitability of Mars and detecting possible life, depends on knowledge of whether combined environmental stresses experienced on Mars are compatible with life as we know it and whether such a record of life could ever be detected. Our current ability to make these assessments is hampered by a lack of knowledge of how combined effects of different environmental stresses influence survival and growth of microorganisms. A range of physical extremes can be examined to assess whether Martian environments have been or are habitable. These stresses are: low temperature (in surface and subsurface environments), high salinity and oxidizing compounds (caused by combinations of salts on Mars, including chlorides, sulfates, perchlorates and the presence of oxidants in soil), aridity (during periods of transient water activity either in the past or present), low availability of nutrients (possibly in all locations), high ionizing radiation (particularly in surface environments on Mars, although these stresses would be less important for deep subsurface life) and anoxic conditions in all Martian environments [3–5]. Some of these factors (e.g. perchlorate and UV-radiation) even strengthen each other mutually in terms of hostility to life [6].

Past attempts to investigate the response of organisms to Martian extremes have generally focused on aerobic organisms including spores of *Bacillus subtilis*, vegetative aerobic halophiles and some phototrophs [7–10]. However, Mars is an anoxic planet and a question that remains unanswered is how anaerobic microorganisms survive under stresses associated with present-day Martian conditions. To address this question, we need to subject highly adaptable anaerobic microorganisms found in diverse environments on the Earth to Martian conditions to ascertain whether characteristics that allow for persistence in extreme physicochemical conditions on Earth would allow these organisms to survive on Mars. This is especially the case for microorganisms that have been accidentally deposited via robotic missions on Mars known as forward contamination. In the context of planetary protection, especially in the case of a mission to Mars, the microbial communities residing in spacecraft assembly clean rooms and on the spacecraft itself were analyzed and their tolerance to Martian conditions was tested. However, their focus was mostly laid on aerobes and single stresses [11–15].

This paper describes the stress response of *Yersinia intermedia* MASE-LG-1 a facultatively anaerobic organism isolated from an extreme Icelandic environment in the framework of MASE (Mars Analogues for Space Exploration) [16]. The following stresses relevant to the Martian environment were tested to advance our knowledge of survivability of a facultatively anaerobic model organism: desiccation, ionizing radiation (X-rays), low pressure, simulated Martian atmosphere, salts and oxidizing compounds, high / low pH, and temperature. In particular, we were interested in the effects of combined environmental stresses, for which data on microbial responses are poorly developed or absent. We report these results and discuss implications for the survival of known anaerobic microorganisms on Mars.

This study will advance our general understanding of the conditions for anaerobic microorganisms on Mars and has implications for the survival of microorganisms accidently deposited on Mars in future robotic or human missions and whether they would survive.

## Materials and methods

### Sampling, enrichment and purification

The sampling site, Lake Grænavatn (63 53.07' N, 22 3.70' W, Iceland), is a restricted area and the sampling campaign was organized and accompanied by the MASE-cooperation partner Viggo T. Marteinsson (MATIS—Prokaria, Reykjavík, Iceland). No protected species were sampled.

*Y*. *intermedia* MASE-LG-1 was isolated from this Icelandic field site (Lake Grænavatn). The lake is a maar type explosion crater, about 35 km south west of Reykjavík in the Reykjanesfólkvangur National Park [17]. Lake Grænavatn is about 45 m deep, approximately 360 m × 260 m in size and hydrothermal activity can be found on the bottom of the lake [17]. The water temperature at the sampling sites were approximately 4°C with a pH-value of around 2. To maintain an oxygen free environment, sampling was performed under anoxic conditions. A determination of the oxygen level at the sampling site was not performed. The samples were taken from the waterside of the lake with a sterile scoop attached to the end of a 1.25 m long pole. Thus, a depth of 30 to 40 cm below the water level was reachable. Water and aqueous / sediment samples were transferred in 100 ml Duran glass flasks. To maintain an anoxic environment, the samples were immediately reduced by injecting a reducing agent (1 ml cysteine-HCl of a 2.5% (w/v) solution) and sealed gastight. Subsequently, microbial enrichments were obtained using the specifically designed MASE I medium, a minimal medium with the addition of various supplements.

MASE I medium contains per liter: NH_4_Cl 0.5 g, NaHCO_3_ 0.2 g, NaH_2_PO_4_, 0.06 g, 10 x Wolfe’s minerals 1 ml [18], 10 x Wolfe’s vitamins 1 ml [16], cysteine-HCl 0.5 g. The medium was portioned (20 ml each) into 120 ml serum bottles and gassed with 1 bar N_2_/CO_2_ (80/20 vol/vol) [19]. Prior to inoculation, the medium was supplemented with 0.01% (w/v) KNO_3_, 0.01% (w/v) C-Org-Mix (C-Org-Mix stock solution contains per 100 ml: yeast extract 1 g, peptone 1 g, brain heart infusion 1 g, meat extract 1 g, 10 x Wolfe’s Vitamins 100 μl). The pH of the MASE I medium was adjusted to 7.0. The incubation was carried out at 30°C and on a shaker at 50 rpm. Strain MASE-LG-1 was enriched and purified in MASE I medium.

### Microscopic observations of the microbial isolate

For electron microscopy, a fresh culture was fixed in paraformaldehyde (final concentration 2.5%), washed three times for 15 min in sodium phosphate buffer (pH 7.4) and filtered onto a 0.2 μm filter. For scanning electron microscopy (SEM), filters were placed into microporous ceramic capsules for dehydration using increasing ethanol / distilled water solutions (25%, 50%, 75% ethanol 15 min each, and 100% ethanol 1 h) followed by a series of ethanol / acetone treatments (25%, 50%, 75% acetone 15 min each, and 100% acetone 1 h). Samples were gold coated and finally observed and analyzed with a Hitachi S4500 Field Emission Gun Scanning Electron Microscope, equipped with an EDX detector (Oxford Instruments). For transmission electron microscopy (TEM), contrast was increased by osmium tetroxide coloration using an OsO_4_ 4% aqueous solution. The upper part of the filter was gold-coated prior to dehydration in order to distinguish the upper and the lower parts of the filters during TEM observations. Dehydration baths for TEM and SEM were identical. For ultrathin sectioning, the samples were prepared by embedding in TAAB 812 Resin (TAAB Laboratories), with a series of acetone / resin mixtures in the proportions 3:1, 1:1, and 3:1, followed by one bath in 100% resin (overnight). The resin blocs were finally left to harden at 60°C in a last 100% resin bath. Ultrathin sections were made using a diatom diamond knife mounted on a Reichert ultra-microtome and placed on copper TEM grids. Observations and analysis were made with a Philips CM20 Transmission Electron Microscope, equipped with an EDX detector.

### Taxonomic classification of the microbial isolate

For phylogenetic identification, gDNA was isolated by the XS buffer method [20]. DNA was amplified by PCR using a 16S rRNA gene primer pair: forward 9Bf and reverse 1406uR [21, 22]. PCR products were purified (PCR purification kit, Quiagen) and Sanger sequenced (GeneArt®). Subsequently, the sequence was submitted to GenBank using Sequin 12.30 (www.ncbi.nlm.nih.gov/Sequin/). The sequence is publicly available (GenBank accession number KX964607). Phylogenetic classification was assessed via BioEdit [23], MEGA6 [24] and MALDI-TOF (following protocol 3 as described previously [25]). To identify the strain at species level two physiological test series, both inoculated with a fresh culture and prepared according to the manufacturer’s instruction were applied (Api® 20A, Rapid ID 32A; bioMérieux).

### Determination of optimal growth conditions

All growth experiments were carried out in triplicates and microbial growth was determined by measuring changes in the optical density at 600 nm. Tested temperatures were 4°C, 25°C, 30°C, 35°C, and 40°C. The pH tests (pH 3 –pH 9 at increments of pH 1) were carried out at optimal growth temperature (T_opt._: 30°C). Sterile anoxic stock solutions of 2 M HCl (acidic pH) and 2 M NaOH (alkaline pH) were used to adjust the pH of the medium. At optimal growth conditions, a growth curve was determined by direct cell counting using a Thoma counting chamber.

### Stress tests and determination of the survival rate

Determination of the survival rate and enumeration of cultivable cells was achieved by the most probable number (MPN) technique via dilution series with ten-fold dilution steps [26]. If not indicated differently, MPN dilution series and stress tests were performed with cells grown in anoxic MASE I medium (Table 1). All experiments were repeated independently at least three times, representing biological replicas. The plotted data represent mean values with standard deviations. Significance was tested by ANOVA analysis of variance. *P*-values < 0.05 were considered statistically significant and were marked by an asterisk within the figures.

**Table 1:** Overview of performed single and combined stress tests with strain MASE-LG-1.

**Single stress**	**Conditions**
Desiccation	≤ 6 month
X-rays	≤ 1000 Gy
Nutrient limitation	Dilution of medium and nutrients (1:10 / 1:50)
Perchlorates	15 min, ≤ 3 M
Osmotic stress (NaCl)	15 min, ≤ 6 M
H_2_O_2_	15 min, ≤ 1 M
Temperature	-86°C—+90°C
**Combined stresses**	**Conditions**
Desiccation and vacuum	≤ 1 month
Desiccation and Mars atmosphere	≤ 1 month
Desiccation and X-rays	1 day; ≤ 800 Gy
X-rays and oxygen	≤ 800 Gy; 21% O_2_
X-rays and desiccation	≤ 800 Gy; 1 day
Nutrient limitation and desiccation	1:10 / 1:50; 1day
Nutrient limitation and X-rays	1:10 / 1:50; ≤ 600 Gy
Perchlorate and desiccation	0.5% / 1%; 1 day
Perchlorate and X-rays	0.5% / 1%; ≤ 700 Gy
NaCl and desiccation	≤ 6 M (15 min); 1 day

The survival rate (S) was calculated as relative survival after cell damaging treatment (N) compared to the non-treated control (N_0_) (S = N/N_0_). D_10_-values were only determined after irradiation. The D_10_-values are indicating the dosage in Gy which reduces the survival rate by one order of magnitude were calculated from the regression lines of the exponential slopes of the survival curves [27].

#### Desiccation experiments and exposure to vacuum or Martian atmosphere

Cells were cultivated under optimal growth conditions until stationary growth phase was reached (~ 24 h). Desiccation experiments were performed as described earlier [28]. One milliliter of culture (equivalent to 10^7^ cells) was spread evenly on sterile glass slides or quartz discs and dried under oxic laboratory conditions (room temperature, relative humidity 33 ± 3.5%) or under anoxic conditions in an anaerobic chamber (Coy Laboratory Products Inc.; [O_2_] < 5 ppm, relative humidity 13 ± 0.5%).

**Fig 1:**
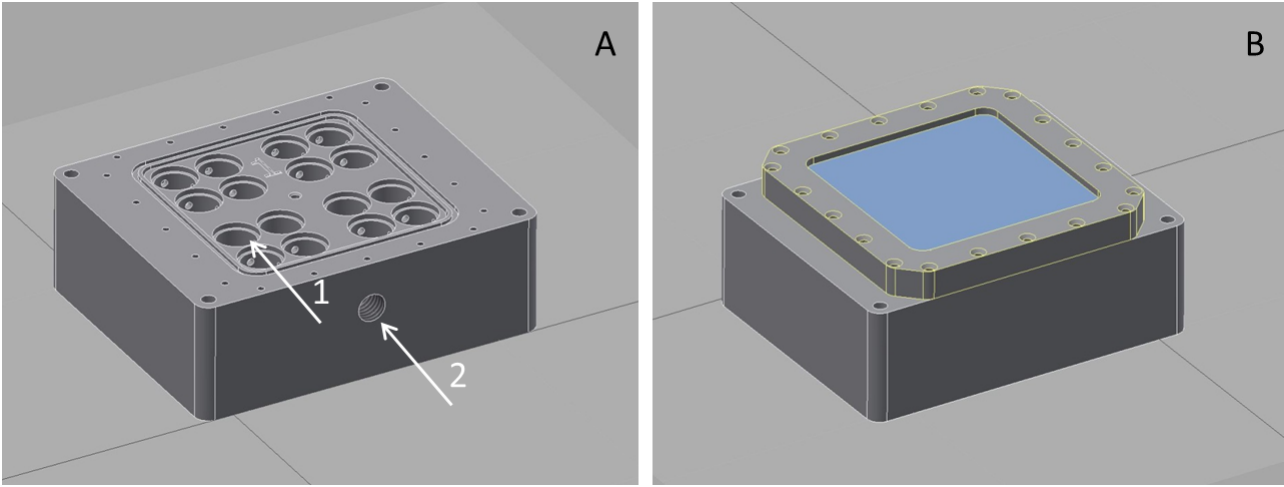
The Trex-Box. The desiccated cells on quartz glass discs can be positioned (arrow 1) inside the open Trex-Box. The box can be connected to various pumping systems (arrow 2) (A). Closed Trex-Box is ready for exposure to combined stresses e.g. radiation and vacuum (B).

Exposure to vacuum or Martian atmosphere was carried out using a newly designed transport and exposure box (Trex-Box) (Fig 1). The Trex-Box is a gastight closable stainless steel box (13.5 cm long, 13.5 cm wide, 5.0 cm high) with one borehole where various pumping systems can be connected which allows for the regulation of the conditions inside the box. The box contains three layers of sample carriers. Each sample carrier has space enough for 16 separate samples (Fig 1A). Desiccated cells on quartz discs were transferred into the Trex-Box under anoxic conditions and were exposed subsequently to low pressure or ionizing radiation.

High vacuum (5 x 10^−5^ Pa) was produced by a pumping system (Rotary vane pump DUO 035 and HIPace 700; Pfeiffer Vacuum GmbH) and continuously monitored (TPG 262 Full Range Gauge; Pfeiffer Vacuum GmbH) during exposure. For simulating the Martian atmosphere, a Mars-like gas composition (2.7% N_2_, 1.6% Ar, 0.15% O_2_ in CO_2_ vol/vol) was used at a pressure of 10^3^ Pa (Fig 1B).

For exposure to ionizing radiation, desiccated cells on glass slides were transferred into gastight closable glass vessels which are normally used for HPLC measurements. Subsequently, the dried cells on glass slides were exposed to X-rays under anoxic conditions.

#### Irradiation experiments

Exposure to ionizing radiation was carried out as described in earlier studies [29]. Cell cultures (in liquid suspension) in HPLC vessels or desiccated cells on glass slides were used. Irradiation was carried out with the X-ray source Gulmay RS 225A (Gulmay Medical Ltd.) at 200 kV and 15 mA. The cells were irradiated at a distance of 19.5 cm below the X-ray source with 20 Gy/min ± 5 Gy/min up to 1000 Gy. The dose rate was measured with a UNIDOS dosimeter (PTW Freiburg GmbH). All irradiation experiments were performed at room temperature.

#### Exposure to Mars relevant chemical compounds

Different concentrations of salts (NaCl, MgCl_2_, CaCl_2_), Martian relevant concentrations of perchlorates (NaClO_4_, Mg(ClO_4_)_2_, Ca(ClO_4_)_2_ [30]) and hydrogen peroxide (H_2_O_2_) were added to an overnight culture grown at optimal conditions (Table 1). After 15 minutes of incubation to these compounds, exposure was terminated by an experimentally feasible high dilution step (1:400) and the MPN assay was applied to determine the survival rate.

#### Exposure to different levels of nutrient availability

To test if the cells are able to grow under nutrient limitation, they were cultivated in medium with a lower content of ingredients (diluted medium). All salts and added supplements were scaled down to 1:10 or 1:50 compared to standard cultivation medium.

#### Combined stresses

To investigate whether stresses act in a synergistic, antagonistic or additive way, several combinations of single stresses were tested (Table 1). To avoid any cellular repair effects, exposure to the combined stressors was done in the shortest time possible.

## Results

Six different bacterial strains, growing under organism specific conditions, were isolated from Lake Grænavatn [16]. One of the strains, namely MASE-LG-1 will be described in detail.

### Strain MASE-LG-1 is affiliated to the genus *Yersinia*

**Fig 2:**
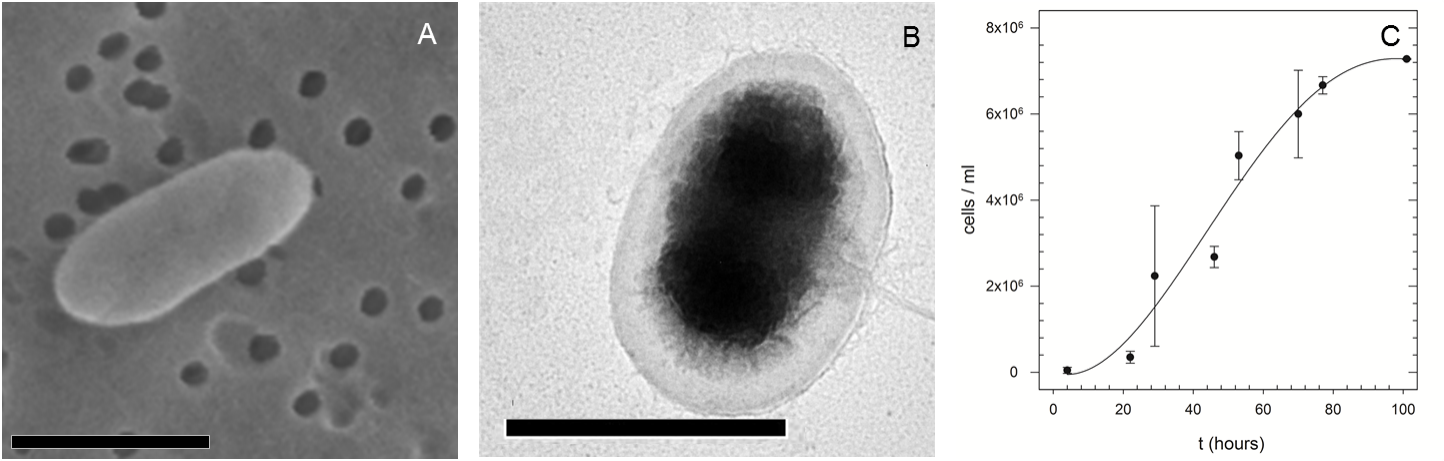
Microscopic images of *Y*. *intermedia* MASE-LG-1. SEM (A) and TEM (B) images of *Y*. *intermedia* MASE-LG-1 and growth curve (C) of *Y*. *intermedia* MASE-LG-1 cultivated under optimal anoxic conditions (30°C, pH 7). Growth was determined by direct cell counting using a Thoma counting chamber (n = 3). Bars: 1.0 μm.

Strain MASE-LG-1 is a motile, rod-shaped, gram negative microorganism ≤ 1.5 μm long (Fig 2A and 2B), growing to a maximal cell density of approximately 10^7^ cells per ml in MASE I medium (Fig 2C).

16S rDNA gene sequence and MALDI-TOF (data not shown) analyses indicated that strain MASE-LG-1 is affiliated to the bacterial genus *Yersinia* (phylum Proteobacteria; S1 Fig). The closest relatives were *Y*. *intermedia*^T^ DSM 18517 [31] and *Yersinia aldovae*^T^ DSM 18303 [32]. Biochemical identifications (API and Rapid test; S1 Table) identified the isolate MASE-LG-1 as *Y*. *intermedia*. Isolation of putative plasmids revealed that *Y*. *intermedia* MASE-LG-1 does not possess any plasmid(s).

*Y*. *intermedia* MASE-LG-1 has been deposited at the Leibniz Institute DSMZ-German Collection of Microorganisms and Cell Cultures and is listed under the DSMZ Acc. No. DSM 102845.

### Evaluation of optimum growth conditions

The temperature range for growth was found to be 25°C– 35°C (optimum of 30°C). No growth occurred at 4°C and above 40°C. The pH range for growth was 5–8 with an optimum of pH 7. Under optimal conditions a generation time of 5 hours and 18 minutes was determined (Fig 2C).

### *Y*. *intermedia* MASE-LG-1 survives prolonged periods of water loss and exposure to ionizing radiation

**Fig 3:**
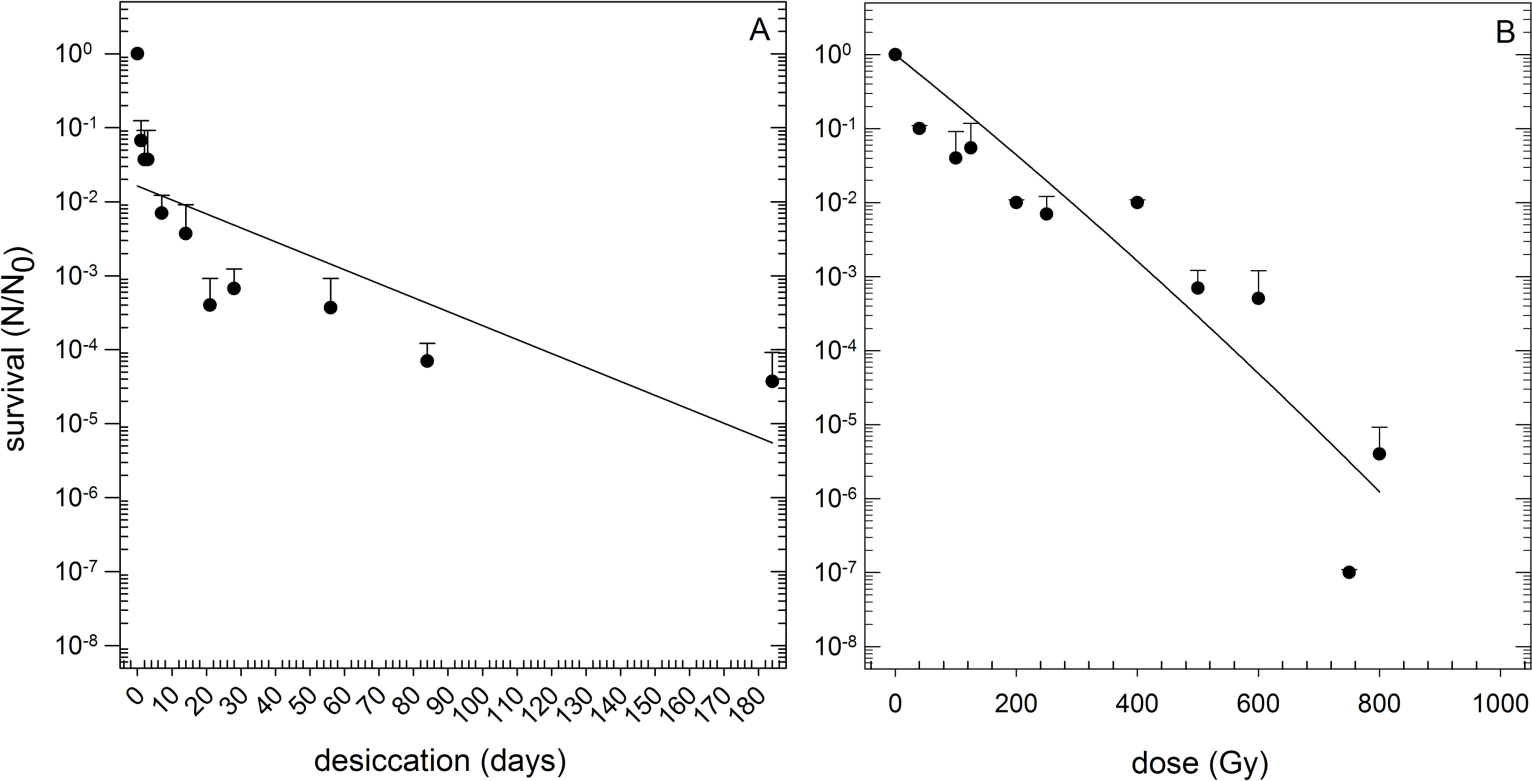
Survival after desiccation and radiation. Survival of *Y*. *intermedia* MASE-LG-1 after anoxic desiccation (A) and after exposure to ionizing radiation (B). N_0:_ Viable cells without desiccation / irradiation, N: Viable cells after desiccation / irradiation (n = 3). (A) Cells were applied on glass slides and dried under anoxic conditions up to 190 days. (B) Cells were exposed to ionizing radiation up to 1000 Gy in liquid culture medium under anoxic conditions.

**Fig 4:**
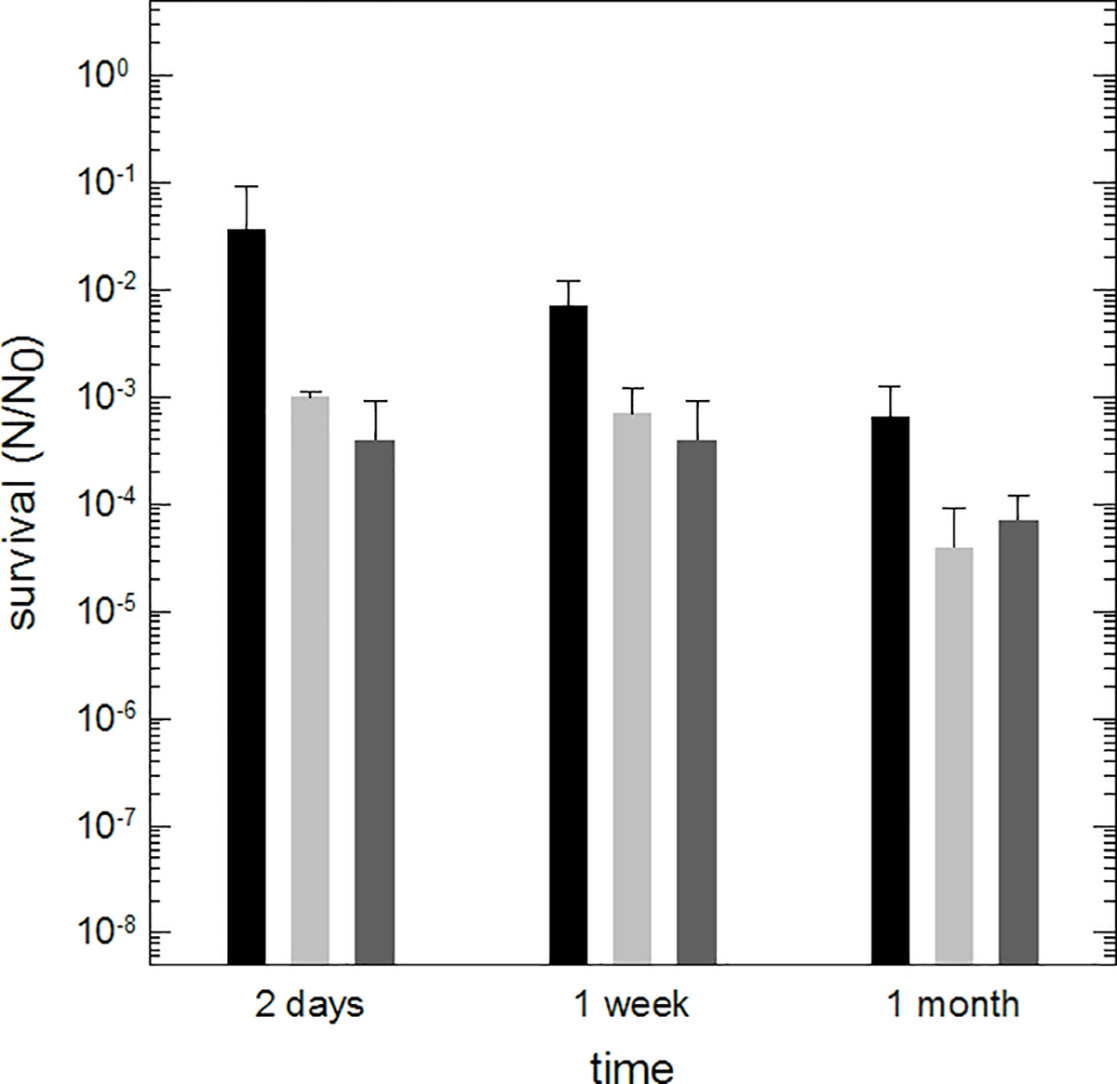
Survival of *Y*. *intermedia* MASE-LG-1 after exposure to desiccation, vacuum and Martian atmosphere. N_0_: viable cells without desiccation / exposure to vacuum, N: viable cells after desiccation / exposure to vacuum (n = 3). Black: Cells were desiccated on glass slides under anoxic conditions. Light grey: Cells were desiccated on quartz discs under anoxic conditions and exposed to vacuum (10^−5^ Pa) within the Trex-Box. Dark grey: Cells were desiccated on quartz discs under anoxic conditions and exposed to Martian atmosphere (Mars gas at a pressure of 10^−3^ Pa) within the Trex-Box.

*Y*. *intermedia* MASE-LG-1 possesses the ability to survive prolonged periods of desiccation under anoxic conditions. The survival rate decreased rapidly after one day of desiccation (S (24 h) = 6.7 x 10^−2^, Fig 3A). After the first 24 h, the survival rate declined more slowly to approximately one order of magnitude per month. After three months of desiccation, the survival was reduced by four orders of magnitude (S (84 days) = 7 x 10^−5^). Cells dried under anoxic conditions were exposed to vacuum representing an extreme form of desiccation and Martian atmosphere. Exposure to vacuum (10^−5^ Pa) and to Martian atmosphere (Mars gas at a pressure of 10^−3^ Pa) led to an additional reduction of survival of one to two orders of magnitude lower than that observed after anoxic desiccation under one terrestrial atmosphere (Fig 4).

By contrast, the strain was sensitive to ionizing radiation. After exposure to 150 Gy, detrimental effects on survivability were visible leading to an overall reduction of more than one order of magnitude (Fig 3B). After exposure to 750 Gy, only marginal survival was observed and after exposure to 1000 Gy, no viable cells were detectable. The calculated D_10_-value was 190 Gy.

### Additive effects of desiccation and ionizing radiation

**Fig 5:**
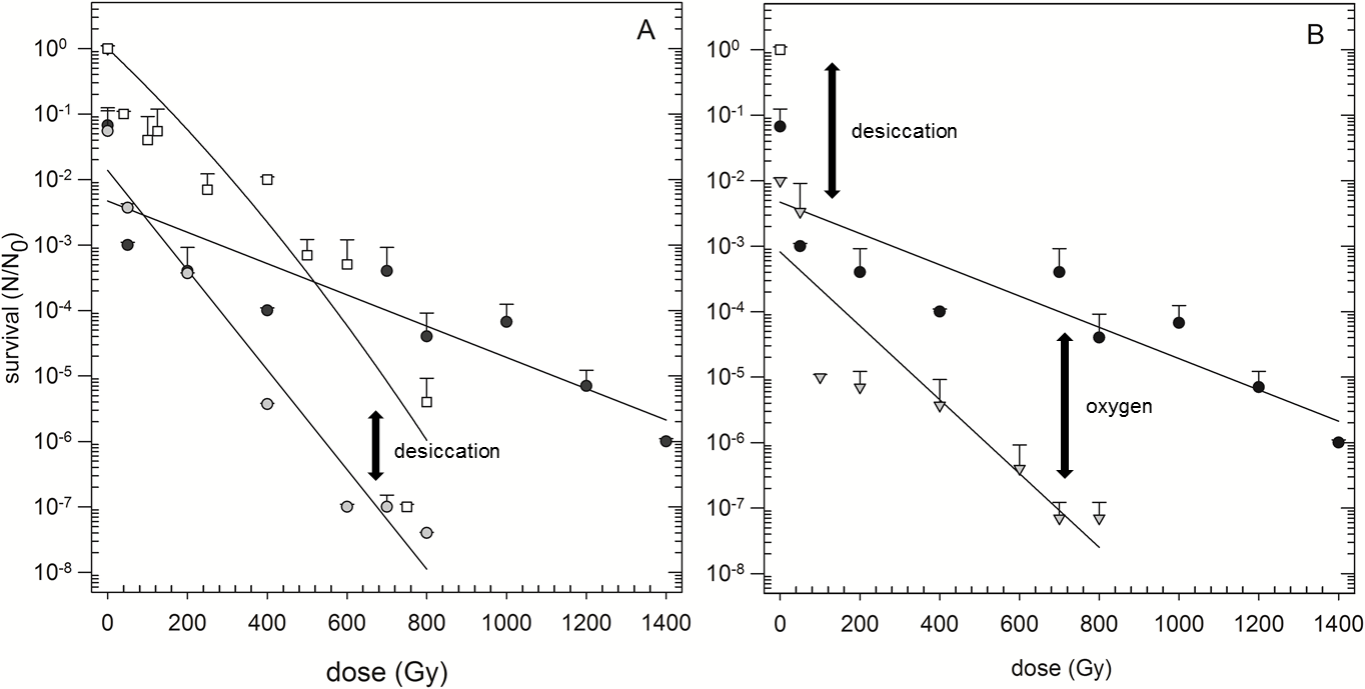
Survival after combined stresses (desiccation and radiation). Survival of *Y*. *intermedia* MASE-LG-1 after desiccation and irradiation in combination (A) and desiccation and irradiation in the presence of oxygen (B). N_0_: Viable cells without desiccation / irradiation. N: Viable cells after desiccation / irradiation (n = 3). (A) White squares: Cells were exposed to ionizing radiation under anoxic conditions in liquid culture medium. Black circles: Cells were desiccated (24 h) under anoxic conditions and subsequently exposed to ionizing radiation under anoxic conditions. Grey circles: Cells were exposed to ionizing radiation under anoxic conditions and subsequently desiccated (24 h) under anoxic conditions. (B) Black circles: Cells were desiccated (24 h) under anoxic conditions and subsequently exposed to ionizing radiation under anoxic conditions. White triangles: Cells were desiccated (24 h) under anoxic conditions and subsequently exposed to ionizing radiation under oxic conditions. White square: Survival of *Y*. *intermedia* MASE-LG-1 without desiccation and irradiation treatment.

In general, the combination of irradiation and desiccation led to additive effects (Fig 5A). However, the manifestation of the additive effect was influenced by the order of the stress treatment. The survival rate of irradiated and desiccated *Y*. *intermedia* MASE-LG-1 shifted to lower survival values compared to cells irradiated in suspension which is likely caused by the effects of desiccation (Fig 5A, arrow marked with “desiccation”). The combination of desiccation and subsequent exposure to irradiation led to a higher survival rate than vice versa (Fig 5A). In a separate experiment, cells were desiccated under anoxic conditions and subsequently irradiated under oxic conditions to determine the effect of oxygen (Fig 5B). When the cells were exposed to oxygen during dry exposure to ionizing radiation the survival rate was lower than after anoxic exposure to ionizing radiation (Fig 5B, arrow marked with “oxygen”). This “oxygen” effect indicates that oxygen functioned as an additional stress parameter.

### Nutrient limitation influences desiccation tolerance

**Fig 6:**
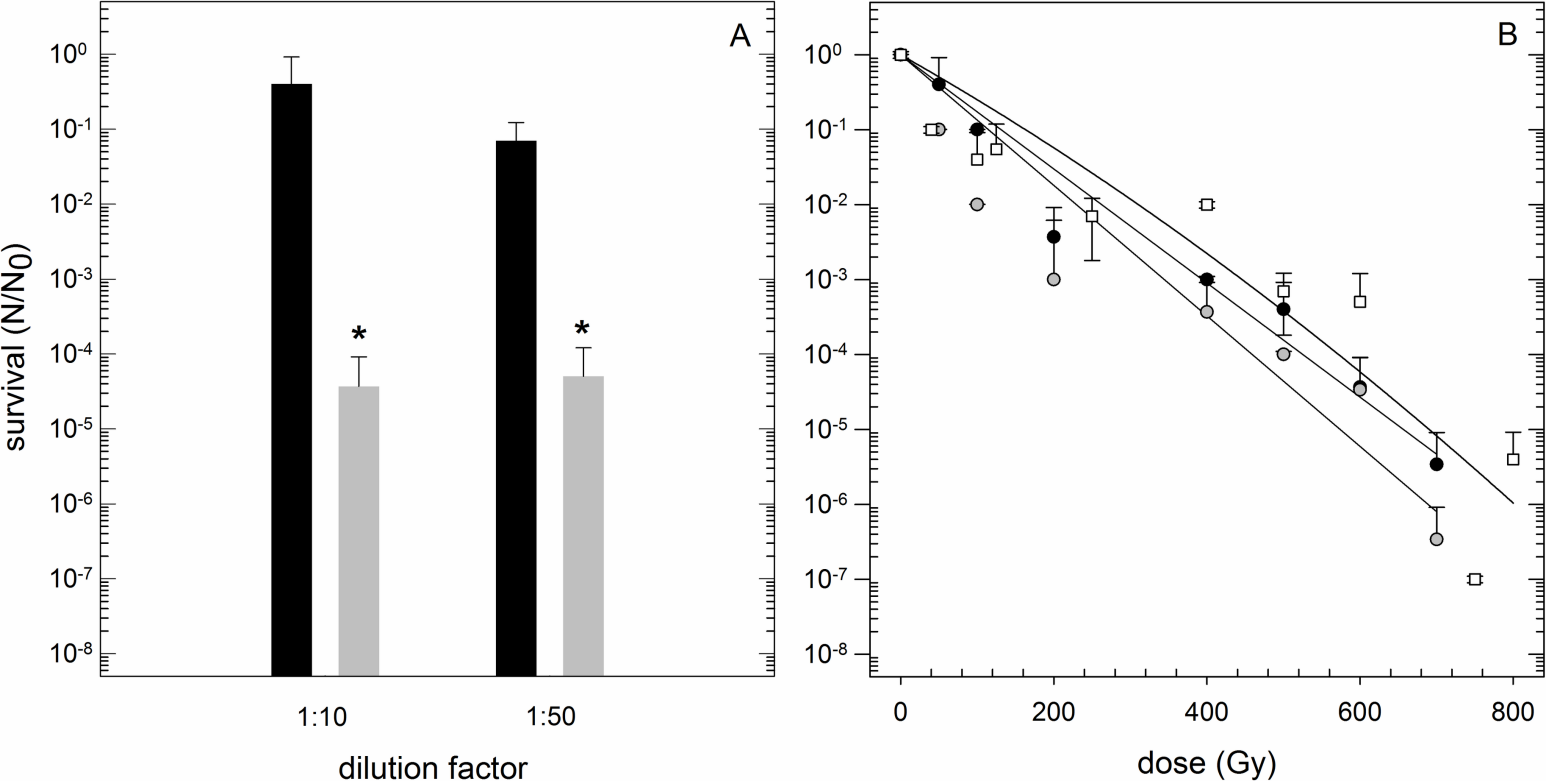
Influence of nutrient limitation. Influence of nutrient limitation on tolerance to desiccation (A) and ionizing radiation (B). N_0_: Viable cells without desiccation / irradiation, N: Viable cells after desiccation / irradiation. Recovery was performed under standard cultivation conditions (n = 3). (A) Black columns: growth in diluted medium after standard cultivation time (24 h). MASE I medium including all supplements was diluted 1:10 / 1:50 before inoculation. Grey columns: survival of *Y*. *intermedia* MASE-LG-1 in diluted medium (1:10 / 1:50) after desiccation (24 h) under anoxic conditions. Asterisks denote significant difference (*p* < 0.05) to the control (desiccation in full medium). (B) Cells grown under a limited set of nutrients were exposed to ionizing radiation up to 800 Gy under anoxic conditions. White squares: MASE I cultivation medium including all supplements without dilution. Black circles: MASE I cultivation medium, including all supplements was diluted 1:10 before inoculation. Grey circles: MASE I cultivation medium, including all supplements was diluted 1:50 before inoculation.

The growth rate in optimal growth conditions was varying with the dilution factor of the medium. A 1:10 dilution of the MASE I medium did not cause a significant change in growth behavior (Fig 6A). Higher dilution (1:50) led to a reduction of the growth rate of one order of magnitude compared to 1:10 diluted medium (Fig 6A). The survival after one day of desiccation was independent of whether the medium was diluted 1:10 or 1:50. In contrast, compared to undiluted medium, the survival rate was significantly lower: S (undiluted, 24 h) = 6.7 x 10^−2^ vs. S (1:10, 24 h) = 3.7 x 10^−5^ (Fig 6A).

The radiation sensitivity slightly increased under nutrient limited conditions (1:50). Cells, grown under nutrient limitation (1:10) showed nearly the same tolerance to ionizing radiation as cells which were grown in undiluted medium (Fig 6B). This was quantified in the calculated D_10_-values: D_10_-value (1:10) 185 Gy, D_10_-value (1:50) 175 Gy compared to the control (190 Gy).

### Survival after exposure to low temperatures, salts and oxidizing compounds

Survival of *Y*. *intermedia* MASE-LG-1 at low temperature (-28°C, -86°C) was tested and the results revealed that the strain can survive low temperatures without added cryoprotectants. After 24 h at -28°C and -86°C no remarkable loss in survivability was observed. In contrast, after one day at +90°C no viable cells were detected (data not shown).

Table 2 summarizes the tolerance of the *Yersinia* strain to potentially cell damaging compounds. *Y*. *intermedia* MASE-LG-1 tolerated different salt concentrations and showed survival after 15 min exposure up to 6.0 M NaCl, 2.0 M CaCl_2_, and 3.0 M MgCl_2_. *Y*. *intermedia* MASE-LG-1 cultures were exposed to oxidizing compounds (hydrogen peroxide and perchlorates) by adding appropriate amounts of stock solutions of the corresponding reagent directly to the grown cultures. Fifteen minutes exposure of oxidizing compounds led to a change in survivability of the strain. For example, no viable cells were detected in hydrogen peroxide concentrations greater than 100 mM. In contrast to the sensitivity against H_2_O_2_, *Y*. *intermedia* MASE-LG-1 was tolerant against different perchlorates. Since both salts, Mg(ClO_4_)_2_ and Ca(ClO_4_)_2_, are formed from divalent cations, the tolerance to ClO_4_^-^ ions up to 2.4 M was comparable.

**Table 2:** Overview of components and concentrations at which growth / survival was detectable after exposure of 15 minutes.

Compound	Concentration
NaCl	≤ 6.0 M
CaCl_2_	≤ 2.0 M
MgCl_2_	≤ 3.0 M
H_2_O_2_	≤ 100 mM
NaClO_4_	≤ 2.4 M
Mg(ClO_4_)_2_	≤ 1.2 M
Ca(ClO_4_)_2_	≤ 1.0 M

### Perchlorates influence desiccation tolerance

**Fig 7:**
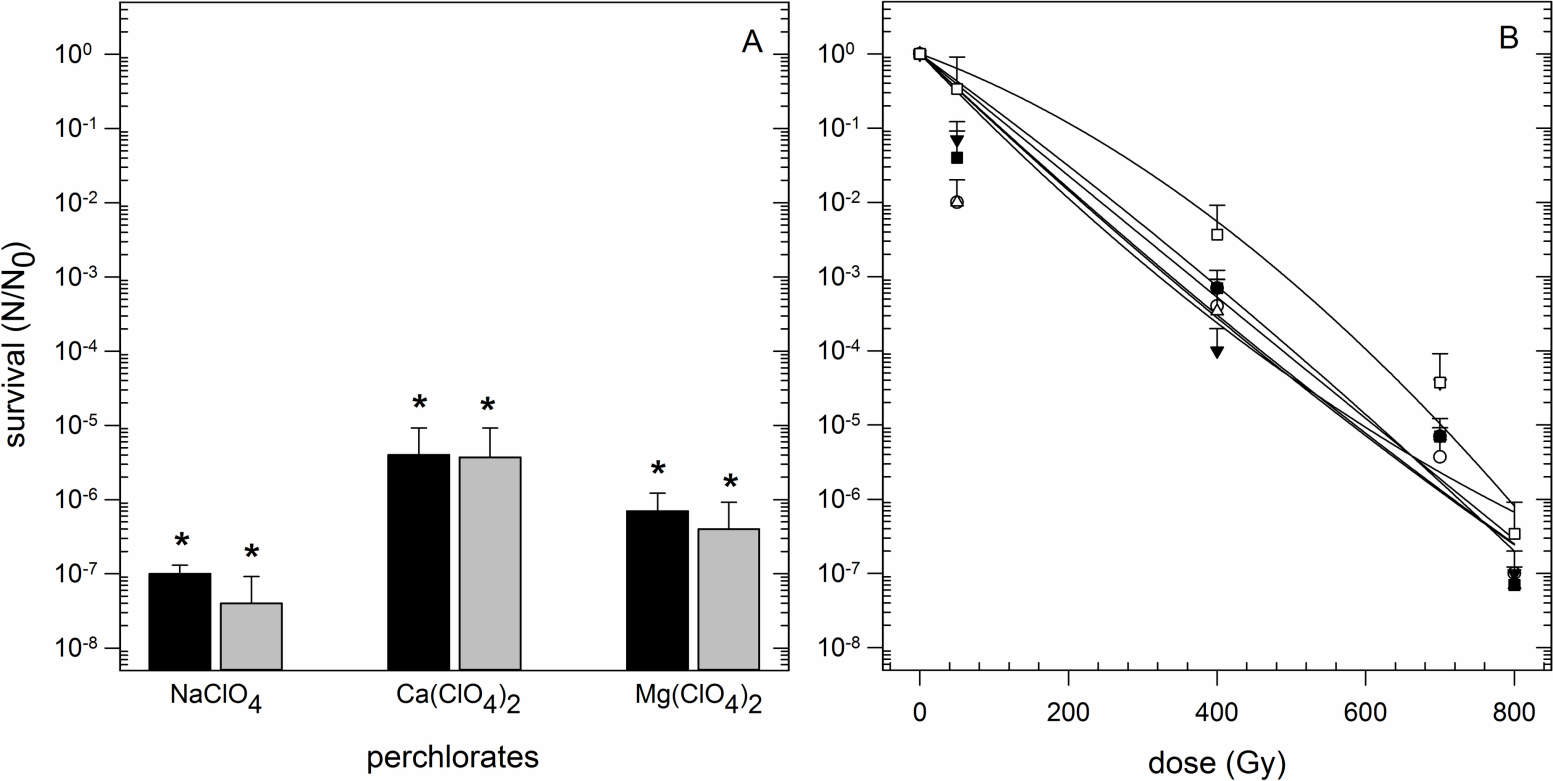
Influence of perchlorates. Influence of perchlorates on tolerance to desiccation (A) and ionizing radiation (B). N_0_: Viable cells without desiccation / irradiation, N: Viable cells after desiccation / irradiation. Recovery was performed under standard cultivation conditions without perchlorate (n = 3). Asterisks denote significant difference (*p* < 0.05) to the control (survival after desiccation without perchlorates). (A) Black columns: Cells were exposed (15 min) to 0.5% perchlorate (0.5% NaClO_4_ = 35.6 mM; 0.5% Ca(Cl_4_)_2_ = 20.9 mM; 0.5% Mg(ClO_4_)_2_ = 22.4 mM) before desiccation treatment (24 h, anoxic conditions). Grey columns: Cells were exposed (15 min) to 1.0% perchlorate (1.0% NaClO_4_ = 71.2 mM; 1.0% Ca(Cl_4_)_2_ = 41.9 mM; 1.0% Mg(ClO_4_)_2_ = 44.8 mM) before desiccation treatment (24 h, anoxic conditions). (B) Cells were exposed (15 min) to the indicated perchlorates before treatment with ionizing radiation up to 800 Gy. Black circles: 0.5% Mg(ClO_4_)_2_; White circle: 1% Mg(ClO_4_)_2_; Black triangle: 0.5% Na(ClO_4_); White triangle: 1% Na(ClO_4_); Black square 0.5% Ca(ClO_4_)_2_; White square: 1% Ca(ClO_4_)_2_.

*Y*. *intermedia* MASE-LG-1 showed a high tolerance against short term exposure to perchlorate ions (2.0–2.4 M; Table 2). Consequently, the combination of perchlorates (in lower concentrations) and subsequent desiccation was investigated. Perchlorates had a significant negative influence on the desiccation tolerance of *Y*. *intermedia* MASE-LG-1 (Fig 7A). In the presence of perchlorates, the survival rate was reduced five to six orders of magnitude than what was observed after desiccation for 24 h without perchlorates. The reduction in the survival rate after desiccation was the same for both 0.5% and 1.0% perchlorates (concentrations for perchlorates in % are w/v). Amongst the particular perchlorates used, NaClO_4_ had the strongest effect on the desiccation tolerance of *Y*. *intermedia* MASE-LG-1.

There was no statistical difference in survival between irradiated cells with and without perchlorates and amongst the two concentrations of the tested perchlorates (0.5% and 1.0%). All regression lines clustered together in a bundle (Fig 7B, ANOVA *p*-value = 0.934). The similarities in the survival curves are also noticeable in the calculated D_10_-values (Table 3).

**Table 3:** Overview of calculated D_10_-values of *Y*. *intermedia* MASE-LG-1 after exposure to ionizing radiation and its combination with other stresses.

Single stress test	Calculated D_10_-value	Combined stress tests	Calculated D_10_-values
X-rays	190 Gy	Nutrient limitation and X-rays 1:10	185 Gy
		Nutrient limitation and X-rays 1:50	175 Gy
		Perchlorate (0.5% (Mg(ClO_4_)_2_) and X-rays	173 Gy
		Perchlorate (1.0% (Mg(ClO_4_)_2_) and X-rays	129 Gy
		Perchlorate (0.5% (NaClO_4_)) and X-rays	93 Gy
		Perchlorate (1.0% (NaClO_4_)) and X-rays	125 Gy
		Perchlorate (0.5% (Ca(ClO_4_)_2_) and X-rays	183 Gy
		Perchlorate (1.0% (Ca(ClO_4_)_2_) and X-rays	189 Gy

## Discussion

Mars is a hostile place for biological processes: anoxic atmosphere, low pressure, a low content of nutrients, presence of cell-damaging compounds, scarcity of liquid water, occurrence of solar and cosmic radiation, and extreme low temperature prevail [4]. Requirements for habitability and microbial life (as we know it), include the availability of traces of liquid water. The lowest water activity measured for organisms from Earth is approximately 0.61 [33]. Additionally, an energy source (chemical or solar), and chemical compounds suitable as nutrients are necessary [5, 34]. The presence of liquid water on the surface in Mars’ early history is well-documented [1–3]. Although liquid water is less prevalent on the surface of Mars today, brines on the surface and potentially in the subsurface is well-reported [35, 36].Various chemical compounds which could function as redox couples can be found and could hypothetically be used by terrestrial organisms [37]. Also perchlorates and other oxidizing substances could be used under anoxic conditions to conserve energy for growth [38, 39]. Despite these factors that support habitability, Mars is also subject to many stresses that are potentially deleterious to life including desiccation, extremes of temperature and pH, ionizing radiation, oxidants and other potentially cell damaging compounds.

Although the response of a wide diversity of aerobic microorganisms has been studied under Martian stresses, systematic studies of anaerobic isolates are lacking. In particular, the response of anaerobic microorganisms to present-day Martian stresses has significant implications for planetary protection considerations which were established to counteract the deposition of terrestrial microbial life forms or signatures thereof onto the surface of Mars. This could be achieved by hardy microorganisms being able to survive the space travel or microorganisms that were accidentally deposited on Mars by robotic or human explorers with the potential to persist on the Martian surface. This process known as forward contamination might negatively impact life detection experiments [40]. We therefore require studies on the survival of cosmopolitan, anaerobic microorganisms that persist in many extreme environments on Earth to understand whether those same capabilities that allow for widespread persistence on Earth would allow for their persistence on Mars.

In this study, we isolated a species of *Yersinia* from an Icelandic environment and chose to use it as a model organism since it represents a highly adaptable, widespread genus found in many anoxic environments on Earth. Various *Y*. *intermedia* strains, the nearest relative to the isolated strain, as well as other *Yersinia* strains are found in freshwater or sewage all over the world [31]. All known strains of *Yersinia* are non-spore forming and for some, especially virulent strains, an ability to survive in aquatic environments and in humidified soils (up to 280 days) is already known [41–43]. It was shown that the virulent *Yersinia pestis* strain CO92 can persist after short term exposure (up to one hour) on different dry surfaces like glass and steel [44].

The persistence of *Yersinia* in many environments raises questions about its metabolic activity in those environments. Initially we attempted cultivation of enrichments at a range of pH conditions, but we were unable to isolate organisms at low pH and low temperatures. The optimal growth conditions of the isolated *Yersinia* strain and the environment bulk pH and temperature of the lake water are different. This discrepancy could be explained with the existence of microhabitats within the lake: e.g. at the bottom of the lake there are volcanic/hydrothermal activities probably due to hot springs [17]. Consequently, micro habitats / niches with optimal growth conditions for the strain could occur. Transcriptomic analyses for another *Y*. *intermedia* strain showed an adaptation mechanism for acid tolerance. This mechanism is especially active if the cells are growing under anoxic conditions [45]. Additionally, there is the discrepancy between the temperature we eventually used for cultivation (30°C) and the natural environment (4°C). The ability to accumulate protective cold shock proteins is known for the genus *Yersinia* [46]. Owing to their pathogenic lifestyle, the genus *Yersinia* is described as highly adaptable [47]. The hostile conditions within the natural environment (i.e. pH extremes, lack of oxygen, and starvation) could result in a pre-adaptation process such as genomic reorganization or even mutation as it is reported for *Escherichia coli* [48] and could lead to the high polyextremotolerance of the strain we isolated. Our findings do not allow us to determine unequivocally the activity of our isolate in the lake. However, its survival and maintenance of viability in such an extreme acidic and cold environment and the known widespread distribution of this anaerobic genus in diverse environments around the world make it an ideal organism with which to assess the capabilities of highly adaptable anaerobic organisms to survive Mars-like conditions. Survival studies focused on Martian UV radiation have been conducted with other widespread distributed organisms like representatives from the genera *Deinococcus* or *Bacillus* [11, 49].

*Y*. *intermedia* MASE-LG-1 was found to be very desiccation tolerant. After 83 days of desiccation the survival rate was lowered only by four orders of magnitude. By comparison, *Hydrogenothermus marinus*, a desiccation-tolerant anaerobic organism also isolated from an aquatic system showed a reduction in its survival rate of 10^−6^ after 112 days of desiccation [28]. Compared with other desiccation-tolerant aerobic organisms, such as *Deinococcus radiodurans*, the survival rate after desiccation was lower. We showed that 0.37% of the *Yersinia* cells survived after a desiccation time of 14 days. The survival rate of *D*. *radiodurans* does not change within the first 100 hours of desiccation and after 400 hours (~ 16 days) 40% survived [50]. The exposure of dried cells to vacuum / Martian atmosphere had no effect on the survival; i.e. once the cells survived the first desiccation step, they were stable with respect to additional desiccation in the form of vacuum and Martian atmosphere. In contrast to our findings, an impact of space vacuum and Martian atmosphere on survival rate of *D*. *radiodurans* has been observed: after exposure to space vacuum (10^−6^ Pa, three days) the survival rate was reduced more than three orders of magnitude compared to desiccated cells [51]. However, if *D*. *radiodurans* is only exposed to Martian atmosphere (7 days) the survival rate was reduced less than one order of magnitude [52].

Research using *D*. *radiodurans*, the model organism in radiation microbiology, suggested that desiccation tolerance and radiation resistance / tolerance are correlated [53, 54]. It has been suggested that the extraordinary high radiation tolerance, which is typical for several *Deinococcus* strains, is a by-product of desiccation tolerance [55, 56]. Additionally, this correlation was shown for different members of the genus *Chroococcidiopsis* [57]. However, this trend is in contrast to the results obtained with *Y*. *intermedia* MASE-LG-1. The isolate was desiccation resistant as described above but was sensitive to ionizing radiation. This was shown in the survival rates as well as in the calculated D_10_-values. For comparison, the D_10_-value given in literature for other vegetative cells such as *E*. *coli* (250 Gy [58]) and *Bacillus pumilus* (210 Gy [59]) are higher than the D_10_-value of our isolate (190 Gy). D_10_-values of other *Yersinia* stains from literature are lower (e.g. *Y*. *enterocolitica* D_10_-value 118 Gy [60]). Interestingly, there is a known dependency between temperature and resistance to radiation: the lower the temperatures are during irradiation treatment, the higher the survival rate is. The D_10_-value for *Y*. *enterocolitica* exposed to ionizing radiation at 0°C is 190 Gy, which is the same D_10_-value as measured for the strain in this study at room temperature. The D_10_-value of *Y*. *enterocolitica* after irradiation treatment at -20°C is 380 Gy; for -76°C even 550 Gy [61].

When the cells were desiccated and subsequently irradiated, they resisted a higher dose of ionizing radiation than in liquid culture. This effect, also known for some halophilic Archaea [62], can be explained by the fact that during desiccation at room temperature, which is near to the optimal growth temperature (30°C), the cells can react actively to the process of desiccation. Intracellular protection mechanisms can take place, e.g. entering a kind of “dormant state” [63]. Furthermore, under desiccation, in conditions with low water availability, there may be lower concentrations of reactive oxygen species caused by interactions of water with ionizing radiation. Therefore, one explanation might be that pre-adaptation to desiccation primed the cells to be less sensitive to other harmful effects e.g. ionizing radiation.

*Y*. *intermedia* MASE-LG-1, having the ability to grow under oxic as well as anoxic conditions, should be capable of resisting the negative effects of an oxygen exposure. It is known that the presence of oxygen during irradiation intensifies the effects of radiation [64, 65]. The strain showed oxygen sensitivity when the irradiation was performed under oxic conditions. As the cells were irradiated in a desiccated form, radiolysis of water and the occurrence of oxygen radicals should be negligible [66]. However, the desiccation step does not completely remove the intracellular H_2_O. This residual water in combination with the applied radiation can lead to an intracellular radical formation by radiolysis. This may explain why the impact was increased with a greater applied dose. When the cells were first irradiated and then desiccated a typical additive effect was observed. The regression line was lowered by two orders of magnitude which was identical to one day of desiccation. The effects of the stresses accumulate additively and the change was the sum of the individual effects [67].

On Mars, only a limited set of nutrients is available and additional cell-damaging components such as high salt concentrations and perchlorates are present on its surface [30, 68, 69]. *Y*. *intermedia* MASE-LG-1 is on the one hand able to grow under a limited set of nutrients, but this nutrient deprivation influenced the desiccation tolerance in a negative way. Likewise, the strain tolerated a short-term exposure to high concentrations of NaCl and perchlorates, but these high salt concentrations and even Mars-like concentrations of perchlorate (0.5% / 1.0%) reduced survival after desiccation as it was already described for *H*. *marinus* [70]. The resistance of the *Yersinia* strain to hydrogen peroxide is lower than that reported for other hydrogen peroxide resistant aerobic organisms and in a comparable range as reported for hydrogen peroxide resistant anaerobic organisms like *Bacteroides fragilis* [71, 72]. *Y*. *intermedia* MASE-LG-1 shows poor survival under exposure to H_2_O_2_ as compared to aerobic *Acinetobacter gyllenbergii* 2P01AA, which is reported to show the highest known tolerance to hydrogen peroxide among Gram-negative and non-spore forming bacteria so far [71]. While *A*. *gyllenbergii* 2P01AA can survive an hour’s exposure to 100 mM H_2_O_2_, after exposure to 320 mM, its survival was reduced by two orders of magnitude and no survival was measurable after exposure to 490 mM. *Y*. *intermedia* MASE-LG-1 can only survive for 15 min in 100 mM H_2_O_2_.

*Y*. *intermedia* MASE-LG-1 exhibits a high tolerance against short term exposure to high amounts of perchlorate ions (up to 2.4 M ≙ 33.7%). There is little known about the upper survival limit under perchlorates exposure of other strains in literature. The cells are often only treated with low concentrations of perchlorate. For example, halophilic archaea are able to grow in 400 mM (≙ 5.6%) sodium perchlorate [73]. The methanogenic archaea *Methanothermobacter wolfeii*, and *Methanosarcina barkeri* were able to survive exposure to 5% and 25% perchlorate (magnesium perchlorate, calcium perchlorate, sodium perchlorate) for variable time periods with *M*. *wolfeii* surviving the 25% (≙ 1.5 M magnesium perchlorate) concentration for longer (72 hours) than *M*. *barkeri* [74].

When compared to real Martian conditions, *Y*. *intermedia* MASE-LG-1 was able to survive some of these Martian conditions for limited periods. On Mars, oxygen is at 0.15% in the thin atmosphere at a pressure of 6 mbar) [3]. The *Y*. *intermedia* MASE-LG-1 was able to withstand Martian atmosphere desiccation for weeks. Low temperatures prevail on Mars’ surface: the average surface temperature is -55°C and at regions at the Martian equator the temperatures sometimes rise above 0°C [3]. The strain was able to tolerate exposures to temperatures down to -86°C without loss in survivability. A putative role of intracellular accumulated cryoprotective compatible solutes cannot be excluded, which has also been proven for *Y*. *enterocolitica* [75]. The radiation dose rate of ionizing radiation on the surface of Mars was measured and calculated to be up to 0.21 mGy per day [76, 77]. Given the results of this study, *Y*. *intermedia* MASE-LG-1 might possibly survive the radiation doses occurring on the Martian surface or in the first centimeter in the Martian soil / regolith for several years or decades [78]. On Mars, other types of radiation occur in galactic cosmic rays and solar energetic particles, which we did not study here.

In conclusion, our data show that even a cosmopolitan, adaptable species of anaerobic microorganism from a genus widely distributed and persistent in many extreme environments on Earth ultimately succumbed to stresses associated with Martian surface conditions. Our data suggest that many similar anaerobic microorganisms might well survive on Mars for short periods of time, but that ultimately they will be killed by Martian conditions. Our data provide a systematic investigation of an anaerobic model microorganism in a variety of Martian extremes. In Martian conditions under which many of these extremes are mitigated (such as subsurface environments) these types of persistent organisms may well have better chances to grow.

## Supporting information

S1 Fig Dendrogram *Y. intermedia* MASE-LG-1. Neighbor-joining tree based on 16S rDNA sequences showing the phylogenetic position of *Y*. *intermedia* MASE-LG-1 related to 14 species of the genus *Yersinia*. DNA sequences were aligned using BioEdit. The dendrogram was constructed by using MEGA6. Bar: 0.01 substitutions per nucleotide position.
(TIF)

S1 Table Results of the API R 20 A and Rapid ID 32 A tests. + *Y*. *intermedia* MASE-LG-1 metabolized the named substance; − *Y*. *intermedia* MASE-LG-1 did not metabolize the named substance.
(DOCX)
