# Supplementary figures and images for: The responses of an anaerobic microorganism, Yersinia intermedia MASE-LG-1 to individual and combined simulated Martian stresses
Source: PLoS One. 2017 Oct 25;12(10):e0185178. doi: 10.1371/journal.pone.0185178 (PMC5656303; doi:10.1371/journal.pone.0185178)

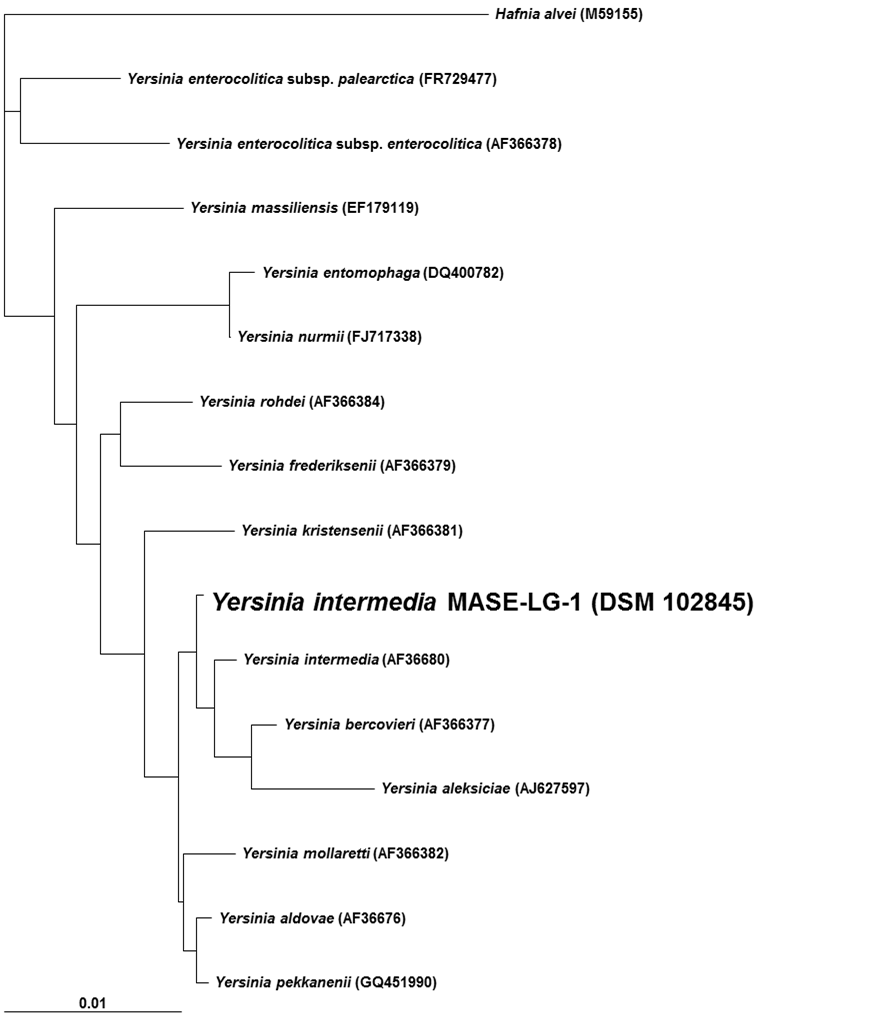

Supplement: S1 Fig — Neighbor-joining tree based on 16S rDNA sequences showing the phylogenetic position of Y. intermedia MASE-LG-1 related to 14 species of the genus Yersinia. DNA sequences were aligned using BioEdit. The dendrogram was constructed by using MEGA6. Bar: 0.01 substitutions per nucleotide position. (TIF) [file pone.0185178.s001.tif]
