# Supplementary material for: The responses of an anaerobic microorganism, Yersinia intermedia MASE-LG-1 to individual and combined simulated Martian stresses
Source: PLoS One. 2017 Oct 25;12(10):e0185178. doi: 10.1371/journal.pone.0185178 (PMC5656303; doi:10.1371/journal.pone.0185178)
Supplement: S1 Table — + Y. intermedia MASE-LG-1 metabolized the named substance; − Y. intermedia MASE-LG-1 did not metabolize the named substance. (DOCX) [file pone.0185178.s002.docx]

**S1 Table. Results of the API R 20 A and Rapid ID 32 A tests.**

| **API R 20 A** | | **Rapid ID 32 A** | |
| --- | --- | --- | --- |
| Indol formation | + | Urea | + |
| Urease | + | 4-nitrophenyl-αD-galactopyranoside | + |
| Glucose | + | 4-nitrophenyl-ßD-galactopyranoside | + |
| Mannitol | + | 4-nitrophenyl-ßD-galactopyranoside-6-phosphate-2CHA | + |
| Saccharose | + | 4-nitrophenyl-ßD-glucopyranoside | + |
| Maltose | + | 4-nitrophenyl-αL-arabinofuropyranoside | + |
| Salicin | + | D-mannose | + |
| Xylose | + | Potassium nitrate | + |
| Arabinose | + | L-tryptophan | + |
| Esculin | + | 2-naphthyl-phosphate | + |
| Glycerol | + | L-arginine-ß-naphthylamide | + |
| Cellobiose | + | L-proline-ß-naphthylamide | + |
| Mannose | + | L-leucyl-L-glycine-ß-naphthylamide | + |
| Raffinose | + | L-phenylalanine-ß-naphthylamide | + |
| Sorbitol | + | L-leucine-ß-naphthylamide | + |
| Rhamnose | + | Pyroglutamic acid ß-naphthylamide | + |
| Trehalose | + | L-tyrosine-ß-naphthylamide | + |
| Catalase | + | L-alanyl-L-alanine-ß-naphthylamide | + |
| Lactose | - | L-glycine-ß-naphthylamide | + |
| Gelatin | - | L-histidine-ß-naphthylamide | + |
| Melezitose | - | L-serine-ß-naphthylamide | + |
|  | | L-arginine | - |
|  |  | 4-nitrophenyl-αD-glucopyranoside | - |
|  |  | 4-nitrophenyl-ßD-glucuronide | - |
|  |  | 4-nitrophenyl-N-acetyl-ßD-glucosaminide | - |
|  |  | D-raffinose | - |
|  |  | Glutamic acid | - |
|  |  | 4-nitrophenyl-αL-fucopyranoside | - |
|  |  | L-glutamyl-L-glutamic acid-ß naphthylamide | - |

+ *Y. intermedia* MASE-LG-1 metabolized the named substance; - *Y. intermedia* MASE-LG-1 did not metabolize the named substance.
